# Supplementary material for: Impact of Coronavirus Disease 2019 on Out-of-Hospital Cardiac Arrest Survival Rate: A Systematic Review with Meta-Analysis
Source: J Clin Med. 2021 Mar 15;10(6):1209. doi: 10.3390/jcm10061209 (PMC8001432; doi:10.3390/jcm10061209)
Supplement: Supplementary file 1 [file jcm-10-01209-s001.pdf]

IMPACT OF CORONAVIRUS DISEASE 2019 ON THE OUT-OF-HOSPITAL CARDIAC ARREST SURVIVAL RATE: A SYSTEMATIC REVIEW WITH META-ANALYSIS

Magdalen J. Borkowska, Milosz J. Jaguszewski, Mariusz Koda, Aleksandra Gasecka, Agnieszka Szarpak, Natasza Gilis-Malinowska, Lukasz Szarpak, Richard Boyer, Krzysztof J. Filipiak, Jacek Smereka

Supplementary File

Content:

Table S1. Methodology characteristics of included studies. 2

Figure S1. Forest plot of patients age in COVID-19 vs. not COVID-19 group........ 4

Figure S2. Forest plot of patients' sex (male) in COVID-19 vs. not COVID-19 group. 4

Figure S3. Forest plot of bystander witnessed in COVID-19 vs. not COVID-19 group. 4

Figure S4. Forest plot of bystander cardiopulmonary resuscitation in COVID-19 vs. not COVID-19 group. 5

Figure S5. Forest plot of advanced life support implementation in COVID-19 vs. not COVID-19 group. 5

Figure S6. Forest plot of first recorded cardiac rhythm in COVID-19 vs. not COVID-19 group 5

Figure S7. Forest plot of mechanical chest compression devices application in COVID-19 vs. not COVID-19 group. 6

Table S1. Methodology characteristics of included studies.

Study	Inclusion criteria	Exclusion criteria	Primary outcome(s)	Findings
Baert et al. 2020	All medical OHCA according to the Utstein template.	Physical indication of death, patients with a known Do Not Attempt Resuscitation (DNAR) order, end of life patients, and traumatic drowning, overdose, asphyxia (external causes) and electrocution OHCA.	the determinants of resuscitation undertaken by bystanders (CPR initiation, type of CPR, use of a defibrillator), secondly, the description of BLS made by the first aid providers (timing, use of ventilation and defibrillator), and lastly, ALS details performed by the MMT (timing, initiation of ALS, administration of epinephrine and tracheal intubation). The other endpoints were return of spontaneous circulation (ROSC) and the survival 30 days after OHCA or at hospital discharge (D30 survival).	During the COVID-19 period, we observed a decrease in CPR initiation regardless of whether patients were suspected of SARS-CoV-2 infection or not. In the current atmosphere, it is important to communicate good resuscitation practices to avoid drastic and lasting reductions in survival rates after an OHCA.
Baldi et al. 2020	All the OHCAs that occurred in these four Provinces in the southern part of the Lombardy Region, in northern Italy, in the first 100 days of epidemic following the first documented case in the Lombardy Region (February 21st , 2020 to May 30th , 2020) and those of the same time frame in 2019 (February 21st , 2019 to May 31st , 2019, to account for the leap year).	NS	NS	Compared to 2019, during the 2020 COVID-19 outbreak we observed a lower attitude of laypeople to start CPR, while resuscitation attempts by BLS and ALS staff were not influenced by suspected/confirmed infection, even at univariable analysis.
Cho et al. 2020	Patients who were aged 18 years or older with OHCA of presumed medical etiology	Patients who did not undergo resuscitative attempts and cases in which cardiac arrest	The treatment and survival outcomes (including prehospital return of spontaneous circulation and neurologic	Responses to the COVID-19 pandemic included changes to current PPE strategies and introduction of isolated resuscitation units; the latter

	and who used the EMS system in Daegu.	occurred in a primary care clinic or long-term care hospital.	outcome at discharge) after cardiac arrest.	intervention reduced the number of unexpected closures and quarantines of emergency resources early on during the COVID-19 outbreak. Given the possibility of future outbreaks, we need to have revised resuscitation strategies and the capacity to commandeer emergency resources for OHCA patients.
Fothergill et al. 2021	All OHCA patients who, during the study periods, received an EMS response from LAS (irrespective of whether a resuscitation attempt was made).	Patients who were successfully resuscitated prior to EMS arrival and so did not receive resuscitation from clinicians.	Survival at 30 days post-arrest.	During the first wave of the COVID-19 pandemic in London, we saw a dramatic rise in the incidence of OHCA, accompanied by a significant reduction in survival. The pattern of increased incidence and mortality closely reflected the rise in confirmed COVID-19 infections in the city.
Sultanian et al. 2021	All cases of OHCA and IHCA registered in the SRCR from 1 January to 20 July 2020.	NS	30-day mortality.	During the pandemic phase, COVID-19 was involved in at least 10% of all OHCAs and 16% of IHCAs, and, among COVID-19 cases, 30-day mortality was increased 3.4-fold in OHCA and 2.3-fold in IHCA.

Legend: ALS = Advanced Life Support; BLS = Basic Life Support; DNAR = Do Not Attempt Resuscitation; NS = Not specified; OHCA = Out-of-hospital cardiac arrest

Study or Subgroup	COVID-19 Mean	SD	Total	Non-COVID-19 Mean	SD	Total	Weight	Mean Difference IV, Random, 95% CI
Baert 2020	67	18	197	69	16	808	13.2%	-2.00 [-4.75, 0.75]
Baldi 2020	77	2.3	125	76.8	2.8	365	40.5%	0.20 [-0.30, 0.70]
Cho 2020	73.3	4.3	10	72.3	3.2	161	13.5%	1.00 [-1.71, 3.71]
Fothergill 2021	70	18	766	71	19	2356	26.0%	-1.00 [-2.49, 0.49]
Sultanian 2021	66.5	18.4	88	70.6	16.4	334	6.8%	-4.10 [-8.33, 0.13]
**Total (95% CI)**			**1186**			**4024**	**100.0%**	**-0.59 [-1.78, 0.61]**

Heterogeneity: $Tau^2 = 0.86$; $Chi^2 = 8.54$, df = 4 (P = 0.07); $I^2 = 53\%$
Test for overall effect: Z = 0.96 (P = 0.34)

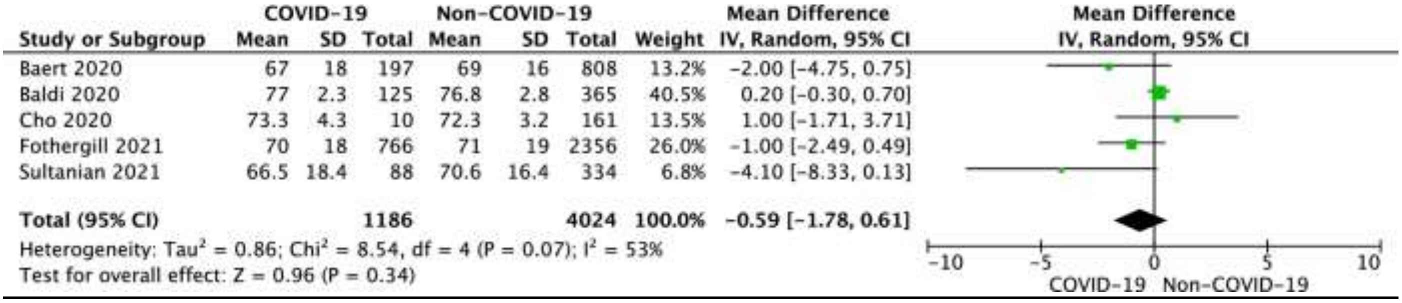

Figure S1. Forest plot of patients age in COVID-19 vs. not COVID-19 group. The center of each square represents the weighted mean differences for individual trials, and the corresponding horizontal line stands for a 95% confidence interval. The diamonds represent pooled results.

Study or Subgroup	COVID-19 Events	Total	Non-COVID-19 Events	Total	Weight	Odds Ratio M-H, Random, 95% CI
Baert 2020	117	197	559	808	25.2%	0.65 [0.47, 0.90]
Baldi 2020	83	125	238	365	20.5%	1.05 [0.69, 1.62]
Cho 2020	4	10	104	161	4.5%	0.37 [0.10, 1.35]
Fothergill 2021	468	766	1371	2356	32.0%	1.13 [0.96, 1.33]
Sultanian 2021	59	88	241	344	17.8%	0.87 [0.53, 1.43]
**Total (95% CI)**		**1186**		**4034**	**100.0%**	**0.88 [0.65, 1.18]**
Total events	731		2513			

Heterogeneity: $Tau^2 = 0.06$; $Chi^2 = 11.47$, df = 4 (P = 0.02); $I^2 = 65\%$
Test for overall effect: Z = 0.85 (P = 0.40)

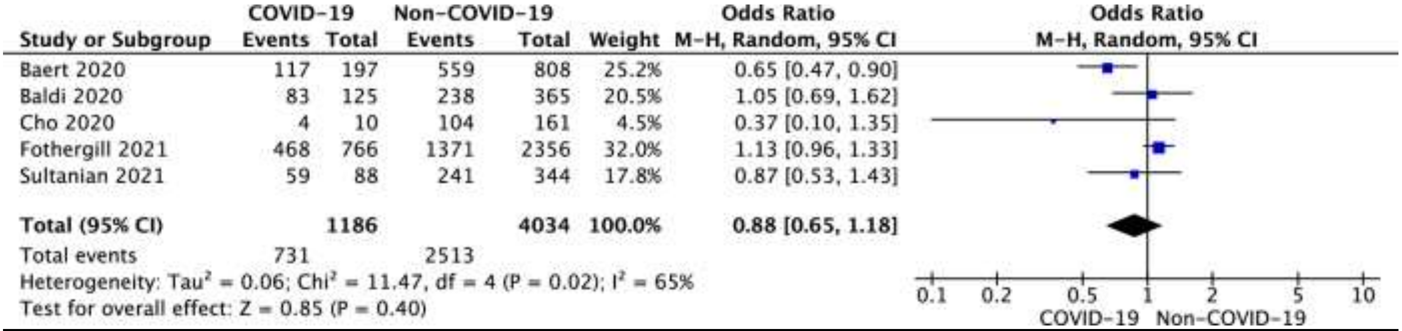

Figure S2. Forest plot of patients' sex (male) in COVID-19 vs. not COVID-19 group. The center of each square represents the weighted odds ratios for individual trials, and the corresponding horizontal line stands for a 95% confidence interval. The diamonds represent pooled results.

Study or Subgroup	COVID-19 Events	Total	Non-COVID-19 Events	Total	Weight	Odds Ratio M-H, Fixed, 95% CI
Baert 2020	126	197	522	808	26.3%	0.97 [0.70, 1.35]
Baldi 2020	39	125	132	365	16.5%	0.80 [0.52, 1.24]
Cho 2020	10	10	120	161	0.2%	7.23 [0.41, 126.15]
Fothergill 2021	216	393	390	742	43.3%	1.10 [0.86, 1.41]
Sultanian 2021	37	88	158	334	13.6%	0.81 [0.50, 1.30]
**Total (95% CI)**		**813**		**2410**	**100.0%**	**0.99 [0.84, 1.17]**
Total events	428		1322			

Heterogeneity: $Chi^2 = 4.22$, df = 4 (P = 0.38); $I^2 = 5\%$
Test for overall effect: Z = 0.08 (P = 0.93)

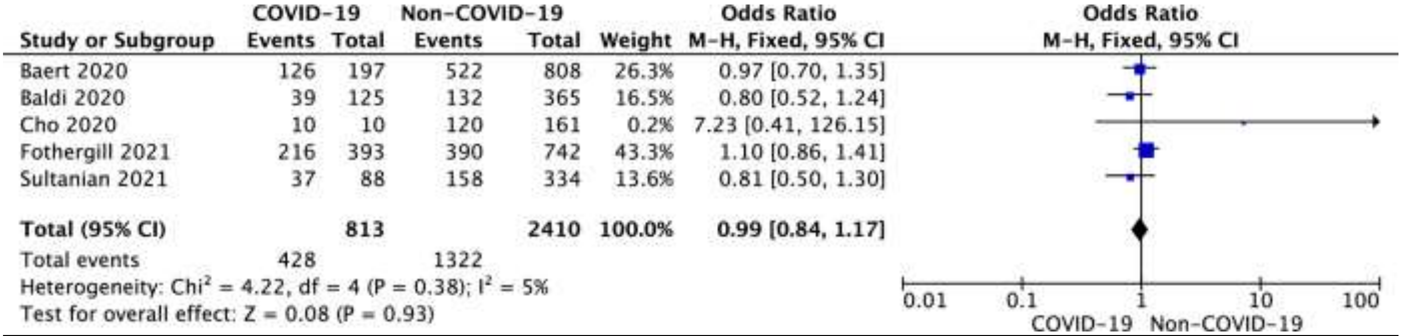

Figure S3. Forest plot of bystander witnessed in COVID-19 vs. not COVID-19 group. The center of each square represents the weighted odds ratios for individual trials, and the corresponding horizontal line stands for a 95% confidence interval. The diamonds represent pooled results.

Study or Subgroup	COVID-19 Events	COVID-19 Total	Non-COVID-19 Events	Non-COVID-19 Total	Weight	Odds Ratio M-H, Random, 95% CI
Baert 2020	99	197	401	808	28.7%	1.03 [0.75, 1.40]
Baldi 2020	13	125	76	365	16.1%	0.44 [0.24, 0.83]
Cho 2020	1	10	57	161	2.3%	0.20 [0.03, 1.64]
Fothergill 2021	257	393	461	742	31.4%	1.15 [0.89, 1.49]
Sultanian 2021	48	88	188	334	21.5%	0.93 [0.58, 1.49]
**Total (95% CI)**		**813**		**2410**	**100.0%**	**0.88 [0.63, 1.22]**
Total events	418		1183			

Heterogeneity: $Tau^2 = 0.07$; $Chi^2 = 10.09$, df = 4 (P = 0.04); $I^2 = 60\%$
Test for overall effect: Z = 0.79 (P = 0.43)

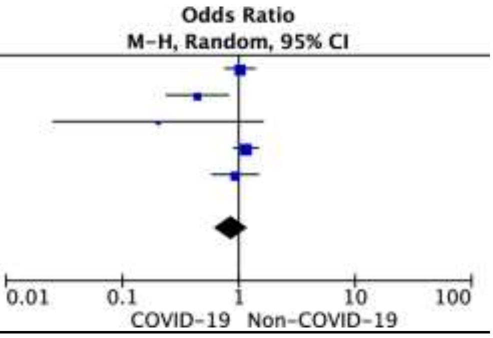

Figure S4. Forest plot of bystander cardiopulmonary resuscitation in COVID-19 vs. not COVID-19 group. The center of each square represents the weighted odds ratios for individual trials, and the corresponding horizontal line stands for a 95% confidence interval. The diamonds represent pooled results.

Study or Subgroup	COVID-19 Events	COVID-19 Total	Non-COVID-19 Events	Non-COVID-19 Total	Weight	Odds Ratio M-H, Fixed, 95% CI
Baert 2020	128	197	548	808	65.2%	0.88 [0.63, 1.22]
Baldi 2020	32	125	106	365	34.8%	0.84 [0.53, 1.33]
**Total (95% CI)**		**322**		**1173**	**100.0%**	**0.87 [0.66, 1.13]**
Total events	160		654			

Heterogeneity: $Chi^2 = 0.03$, df = 1 (P = 0.87); $I^2 = 0\%$
Test for overall effect: Z = 1.05 (P = 0.29)

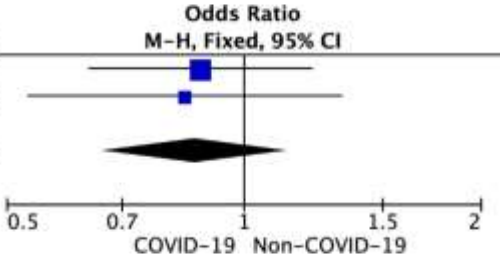

Figure S5. Forest plot of advanced life support implementation in COVID-19 vs. not COVID-19 group. The center of each square represents the weighted odds ratios for individual trials, and the corresponding horizontal line stands for a 95% confidence interval. The diamonds represent pooled results.

Study or Subgroup	COVID-19 Events	COVID-19 Total	Non-COVID-19 Events	Non-COVID-19 Total	Weight	Odds Ratio M-H, Random, 95% CI
**3.6.1 Shockable**						
Baert 2020	8	197	633	806	21.8%	0.01 [0.01, 0.02]
Baldi 2020	8	125	29	365	21.5%	0.79 [0.35, 1.78]
Cho 2020	0	10	10	161	13.0%	0.69 [0.04, 12.55]
Fothergill 2021	24	393	144	742	22.4%	0.27 [0.17, 0.42]
Sultanian 2021	6	80	63	276	21.3%	0.27 [0.11, 0.66]
**Subtotal (95% CI)**		**805**		**2350**	**100.0%**	**0.19 [0.04, 0.96]**
Total events	46		879			
Heterogeneity: $Tau^2 = 2.91$; $Chi^2 = 76.48$, df = 4 (P < 0.00001); $I^2 = 95\%$						
Test for overall effect: Z = 2.01 (P = 0.04)						
**3.6.2 Not shockable**						
Baert 2020	181	197	693	806	35.2%	1.84 [1.07, 3.19]
Baldi 2020	80	125	198	365	46.5%	1.50 [0.99, 2.28]
Sultanian 2021	74	80	213	276	18.4%	3.65 [1.52, 8.78]
**Subtotal (95% CI)**		**402**		**1447**	**100.0%**	**1.90 [1.24, 2.90]**
Total events	335		1104			
Heterogeneity: $Tau^2 = 0.06$; $Chi^2 = 3.26$, df = 2 (P = 0.20); $I^2 = 39\%$						
Test for overall effect: Z = 2.96 (P = 0.003)						

Test for subgroup differences: $Chi^2 = 7.32$, df = 1 (P = 0.007), $I^2 = 86.3\%$

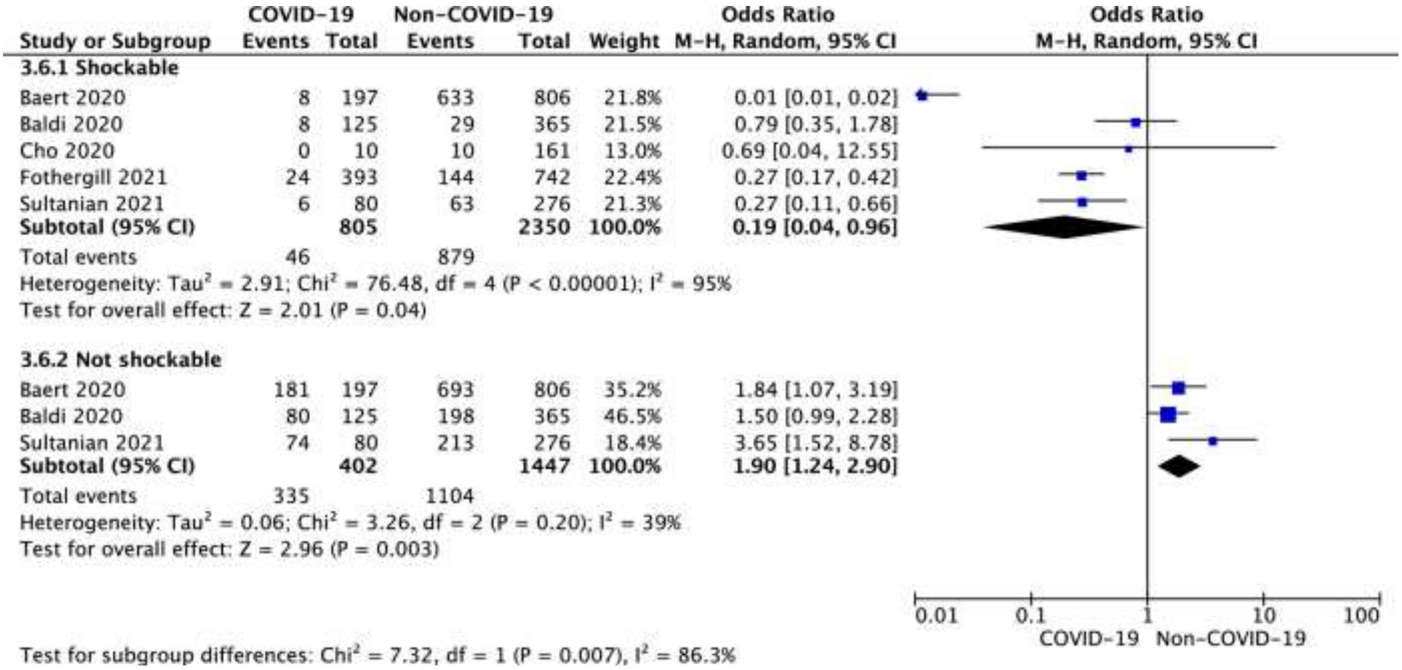

Figure S6. Forest plot of first recorded cardiac rhythm in COVID-19 vs. not COVID-19 group. The center of each square represents the weighted odds ratios for individual trials, and the corresponding horizontal line stands for a 95% confidence interval. The diamonds represent pooled results.

Study or Subgroup	COVID-19 Events	COVID-19 Total	Non-COVID-19 Events	Non-COVID-19 Total	Weight	Odds Ratio M-H, Fixed, 95% CI
Baldi 2020	2	125	7	365	44.1%	0.83 [0.17, 4.06]
Cho 2020	7	10	127	161	55.9%	0.62 [0.15, 2.54]
**Total (95% CI)**		**135**		**526**	**100.0%**	**0.72 [0.25, 2.09]**
Total events	9		134			

Heterogeneity: $Chi^2 = 0.07$, df = 1 (P = 0.79); $I^2 = 0\%$

Test for overall effect: Z = 0.61 (P = 0.54)

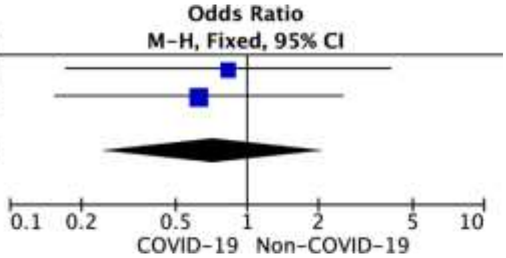

Figure S7. Forest plot of mechanical chest compression devices application in COVID-19 vs. not COVID-19 group. The center of each square represents the weighted odds ratios for individual trials, and the corresponding horizontal line stands for a 95% confidence interval. The diamonds represent pooled results.
